# *catena*-Poly[1,2,2-trimethyl­cyclo­pentane-1,3-diammonium [aluminate(III)-μ-(hydrogen phosphato)-μ-phosphato]]

**DOI:** 10.1107/S1600536812028826

**Published:** 2012-06-30

**Authors:** Li-Li Liang

**Affiliations:** a Department of Chemistry, Bengbu Medical College, Bengbu 233030, People’s Republic of China

## Abstract

In the title compound, {(C_8_H_20_N_2_)[Al(HPO_4_)(PO_4_)]}_*n*_, the Al^III^ atom is coordinated by four O atoms from two HPO_4_^2−^ and two PO_4_^3−^ groups in a distorted tetra­hedral geometry. Each AlO_4_ unit shares four O atoms with four adjacent PO_4_ units, leading to an anionic chain along [100]. The negative charge of the chain is compensated by doubly protonated camphoric amine cations. N—H⋯O hydrogen bonds connect the cations and the anionic chains. O—H⋯O hydrogen bonds are present in the chain.

## Related literature

For the synthesis and applications of chiral inorganic framework materials, see: Viter & Nagornyi (2009 ▶). For information about aluminophosphate chains, see: Jones *et al.* (1990 ▶); Oliver *et al.* (1998 ▶); Williams *et al.* (1997 ▶).
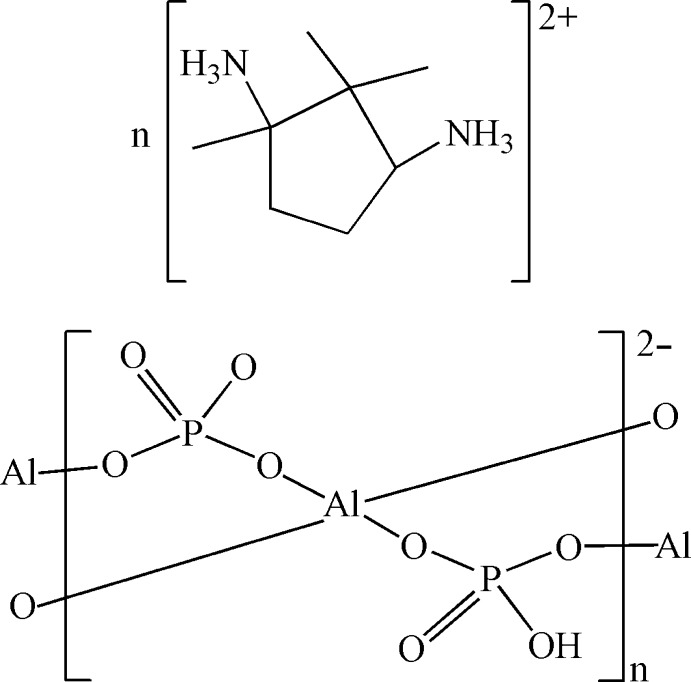


## Experimental

### 

#### Crystal data

(C_8_H_20_N_2_)[Al(HPO_4_)(PO_4_)]
*M* *_r_* = 362.19
Monoclinic, 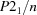

*a* = 8.0102 (10) Å
*b* = 16.862 (2) Å
*c* = 10.5164 (12) Å
β = 93.203 (2)°
*V* = 1418.2 (3) Å^3^
*Z* = 4
Mo *K*α radiation
μ = 0.41 mm^−1^
*T* = 293 K
0.22 × 0.19 × 0.18 mm

#### Data collection

Bruker APEX CCD diffractometer
Absorption correction: multi-scan (*SADABS*; Bruker, 2001 ▶) *T*_min_ = 0.915, *T*_max_ = 0.930
8701 measured reflections
3386 independent reflections
2442 reflections with *I* > 2σ(*I*)
*R*_int_ = 0.078

#### Refinement

*R*[*F* ^2^ > 2σ(*F* ^2^)] = 0.041
*wR*(*F* ^2^) = 0.098
*S* = 1.02
3386 reflections
195 parameters
H-atom parameters constrained
Δρ_max_ = 0.47 e Å^−3^
Δρ_min_ = −0.47 e Å^−3^

### 

Data collection: *SMART* (Bruker, 2007 ▶); cell refinement: *SAINT* (Bruker, 2007 ▶); data reduction: *SAINT*; program(s) used to solve structure: *SHELXS97* (Sheldrick, 2008 ▶); program(s) used to refine structure: *SHELXL97* (Sheldrick, 2008 ▶); molecular graphics: *DIAMOND* (Brandenburg, 1999 ▶); software used to prepare material for publication: *SHELXTL* (Sheldrick, 2008 ▶).

## Supplementary Material

Crystal structure: contains datablock(s) global, I. DOI: 10.1107/S1600536812028826/hy2556sup1.cif

Supplementary material file. DOI: 10.1107/S1600536812028826/hy2556Isup2.mol

Supplementary material file. DOI: 10.1107/S1600536812028826/hy2556Isup3.cdx

Structure factors: contains datablock(s) I. DOI: 10.1107/S1600536812028826/hy2556Isup4.hkl

Supplementary material file. DOI: 10.1107/S1600536812028826/hy2556Isup5.cdx

Additional supplementary materials:  crystallographic information; 3D view; checkCIF report

## Figures and Tables

**Table 1:** Hydrogen-bond geometry (Å, °)

*D*—H⋯*A*	*D*—H	H⋯*A*	*D*⋯*A*	*D*—H⋯*A*
N1—H1*A*⋯O3	0.89	1.87	2.751 (2)	168
N1—H1*B*⋯O5^i^	0.89	2.06	2.910 (3)	158
N1—H1*C*⋯O8^ii^	0.89	1.85	2.709 (3)	162
N2—H2*A*⋯O3^iii^	0.89	2.00	2.858 (2)	160
N2—H2*B*⋯O8^iv^	0.89	2.14	3.010 (2)	167
N2—H2*C*⋯O7^v^	0.89	1.89	2.766 (3)	169
O5—H5*B*⋯O7^vi^	0.96	1.59	2.498 (2)	156

Symmetry codes: (i) 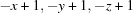; (ii) 
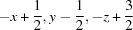
; (iii) 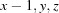; (iv) 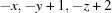; (v) 
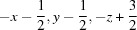
; (vi) 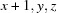.

**Fig. 1.**
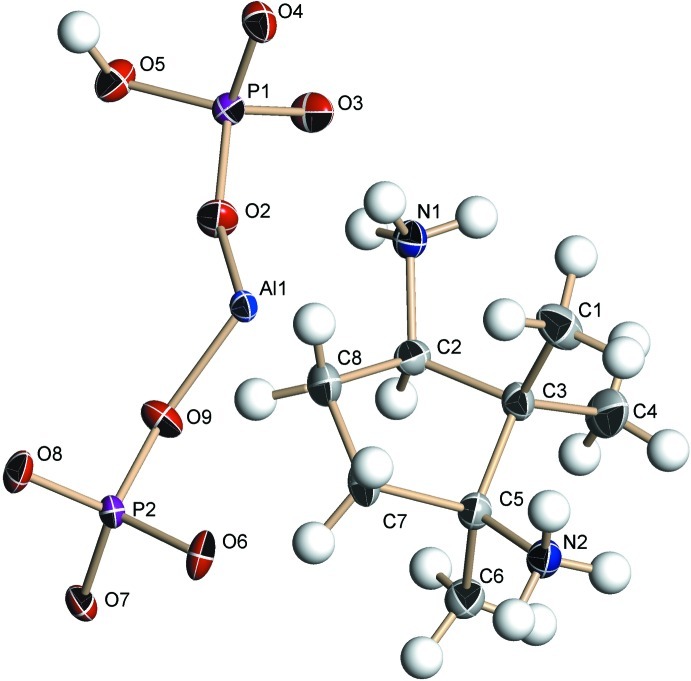
The asymmetric unit of the title compound. Displacement ellipsoids are drawn at the 50% probability level.

**Fig. 2.**
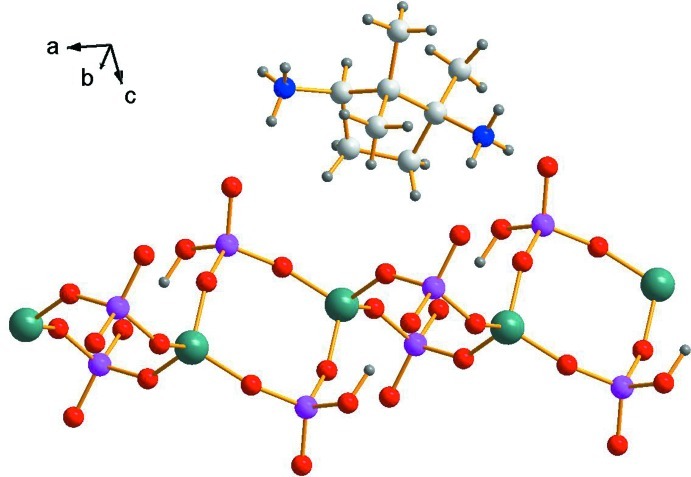
The anionic chain and camphoric amine cation in the title compound.

## supplementary crystallographic information

### Comment

There are considerable interests in the synthesis of chiral inorganic framework materials for their potential applications in separation and catalysis (Viter & Nagornyi, 2009). Our interest is particularly focused on the synthesis of microporous aluminophosphate. We used organic camphoric amine as the template and hydrothermally synthesized the title compound (Fig. 1).

The structure consists of aluminophosphate chains of formula [Al(HPO_4_)(PO_4_)]_n_, running along the *a* axis, and doubly protonated camphoric amine cations (Fig. 2). The chain is constructed from AlO_4_ tetrahedra and PO_4_ tetrahedra. Each AlO_4_ tetrahedron shares four O atoms with adjacent PO_4_ tetrahedra, whereas each PO_4_ tetrahedron shares two O atoms with adjacent AlO_4_ tetrahedra, leaving the other two O atoms terminal. The structure denotes that AlPO-CSC (Corner-Sharing Chain) is one of the fundamental chains in the known aluminophosphate compounds (Jones *et al.*, 1990; Oliver *et al.*, 1998; Williams *et al.*, 1997). The negative charge of the chain is compensated by protonated organic camphoric amine cations, which are connected to the chains through N—H···O hydrogen bonds (Table 1).

### Experimental

A mixture of aluminium isopropoxide (204 mg, 1 mmol), water (720 mg, 40 mmol), 85% H_3_PO_4_ (254 mg, 2.2 mmol), camphoric amine (143 mg, 1 mmol) and HF (13.0 mg, 0.65 mmol) was stirred to form a gel. The gel was sealed in a Teflon-lined stainless-steel autoclave and heated at 180°C for 10 days. Colorless crystals were collected by filtration, washed with distilled water and dried in air.

### Refinement

H atoms on C and N atoms were positioned geometrically and refined as riding atoms, with C—H = 0.98 (CH), 0.97 (CH_2_) and 0.96 (CH_3_) Å and N—H = 0.89 Å and with *U*_iso_(H) = 1.2(1.5 for methyl and ammonium)*U*_eq_(C,N). Hydroxyl H atom was located from a difference Fourier map and refined as riding, with O—H = 0.96 Å and *U*_iso_(H) = 1.5*U*_eq_(O).

### Crystal data

**Table:**

(C_8_H_20_N_2_)[Al(HPO_4_)(PO_4_)]	*F*(000) = 760
*M**_r_* = 362.19	*D*_x_ = 1.696 Mg m^−^^3^
Monoclinic, *P*2_1_/*n*	Mo *K*α radiation, λ = 0.71073 Å
Hall symbol: -P 2yn	Cell parameters from 2335 reflections
*a* = 8.0102 (10) Å	θ = 2.3–22.7°
*b* = 16.862 (2) Å	µ = 0.41 mm^−^^1^
*c* = 10.5164 (12) Å	*T* = 293 K
β = 93.203 (2)°	Block, colourless
*V* = 1418.2 (3) Å^3^	0.22 × 0.19 × 0.18 mm
*Z* = 4	

### Data collection

**Table:**

Bruker APEX CCD diffractometer	3386 independent reflections
Radiation source: fine-focus sealed tube	2442 reflections with *I* > 2σ(*I*)
Graphite monochromator	*R*_int_ = 0.078
φ and ω scans	θ_max_ = 28.2°, θ_min_ = 2.3°
Absorption correction: multi-scan (*SADABS*; Bruker, 2001)	*h* = −10→10
*T*_min_ = 0.915, *T*_max_ = 0.930	*k* = −18→22
8701 measured reflections	*l* = −13→11

### Refinement

**Table:**

Refinement on *F*^2^	Primary atom site location: structure-invariant direct methods
Least-squares matrix: full	Secondary atom site location: difference Fourier map
*R*[*F*^2^ > 2σ(*F*^2^)] = 0.041	Hydrogen site location: inferred from neighbouring sites
*wR*(*F*^2^) = 0.098	H-atom parameters constrained
*S* = 1.02	*w* = 1/[σ^2^(*F*_o_^2^) + (0.0302*P*)^2^] where *P* = (*F*_o_^2^ + 2*F*_c_^2^)/3
3386 reflections	(Δ/σ)_max_ = 0.001
195 parameters	Δρ_max_ = 0.47 e Å^−^^3^
0 restraints	Δρ_min_ = −0.47 e Å^−^^3^

### Special details

**Table:**

Geometry. All e.s.d.'s (except the e.s.d. in the dihedral angle between two l.s. planes) are estimated using the full covariance matrix. The cell e.s.d.'s are taken into account individually in the estimation of e.s.d.'s in distances, angles and torsion angles; correlations between e.s.d.'s in cell parameters are only used when they are defined by crystal symmetry. An approximate (isotropic) treatment of cell e.s.d.'s is used for estimating e.s.d.'s involving l.s. planes.
Refinement. Refinement of *F*^2^ against ALL reflections. The weighted *R*-factor *wR* and goodness of fit *S* are based on *F*^2^, conventional *R*-factors *R* are based on *F*, with *F* set to zero for negative *F*^2^. The threshold expression of *F*^2^ > σ(*F*^2^) is used only for calculating *R*-factors(gt) *etc*. and is not relevant to the choice of reflections for refinement. *R*-factors based on *F*^2^ are statistically about twice as large as those based on *F*, and *R*- factors based on ALL data will be even larger.

### Fractional atomic coordinates and isotropic or equivalent isotropic displacement parameters (Å^2^)

**Table:**

	*x*	*y*	*z*	*U*_iso_*/*U*_eq_	
Al1	0.23800 (9)	0.51205 (4)	0.94544 (6)	0.01494 (17)	
C1	0.1616 (3)	0.26568 (17)	0.7028 (3)	0.0337 (7)	
H1D	0.0959	0.2257	0.7416	0.051*	
H1E	0.1789	0.3094	0.7606	0.051*	
H1F	0.2678	0.2437	0.6833	0.051*	
C2	0.1662 (3)	0.36187 (15)	0.5190 (2)	0.0223 (5)	
H2	0.1159	0.3716	0.4333	0.027*	
C3	0.0691 (3)	0.29490 (14)	0.5794 (2)	0.0214 (5)	
C4	0.0404 (4)	0.22449 (17)	0.4892 (3)	0.0385 (7)	
H4A	−0.0082	0.2429	0.4090	0.058*	
H4B	−0.0340	0.1873	0.5258	0.058*	
H4C	0.1452	0.1991	0.4761	0.058*	
C5	−0.0927 (3)	0.34152 (15)	0.6070 (2)	0.0215 (5)	
C6	−0.2124 (3)	0.34974 (16)	0.4893 (2)	0.0270 (6)	
H6A	−0.2663	0.2998	0.4714	0.041*	
H6B	−0.1509	0.3654	0.4177	0.041*	
H6C	−0.2953	0.3892	0.5049	0.041*	
C7	−0.0302 (3)	0.42256 (15)	0.6572 (2)	0.0253 (6)	
H7A	−0.1081	0.4642	0.6305	0.030*	
H7B	−0.0179	0.4222	0.7495	0.030*	
C8	0.1363 (3)	0.43549 (16)	0.6010 (3)	0.0341 (7)	
H8B	0.2241	0.4408	0.6678	0.041*	
H8A	0.1340	0.4831	0.5491	0.041*	
N1	0.3491 (2)	0.34899 (12)	0.50846 (18)	0.0210 (5)	
H1A	0.4045	0.3681	0.5775	0.031*	
H1B	0.3826	0.3739	0.4397	0.031*	
H1C	0.3695	0.2973	0.5019	0.031*	
N2	−0.1918 (2)	0.30479 (12)	0.70978 (18)	0.0229 (5)	
H2A	−0.2803	0.3350	0.7231	0.034*	
H2B	−0.1277	0.3011	0.7814	0.034*	
H2C	−0.2258	0.2566	0.6854	0.034*	
O2	0.3899 (2)	0.51208 (10)	0.83594 (16)	0.0248 (4)	
O3	0.5465 (2)	0.42016 (10)	0.69810 (15)	0.0258 (4)	
O4	0.6755 (2)	0.46404 (10)	0.90504 (15)	0.0236 (4)	
O5	0.6361 (2)	0.56178 (11)	0.72750 (15)	0.0257 (4)	
H5B	0.7125	0.5872	0.7881	0.039*	
O6	−0.1454 (2)	0.58055 (10)	1.04650 (15)	0.0256 (4)	
O7	−0.1712 (2)	0.65708 (10)	0.84337 (15)	0.0240 (4)	
O8	0.0256 (2)	0.70185 (10)	1.02657 (15)	0.0238 (4)	
O9	0.0880 (2)	0.58103 (10)	0.89830 (16)	0.0246 (4)	
P1	0.56043 (8)	0.48755 (4)	0.78985 (6)	0.01633 (16)	
P2	−0.04921 (8)	0.63267 (4)	0.95434 (6)	0.01681 (16)	

### Atomic displacement parameters (Å^2^)

**Table:**

	*U*^11^	*U*^22^	*U*^33^	*U*^12^	*U*^13^	*U*^23^
Al1	0.0163 (4)	0.0119 (4)	0.0167 (4)	0.0005 (3)	0.0018 (3)	−0.0008 (3)
C1	0.0363 (16)	0.0348 (17)	0.0308 (15)	0.0102 (13)	0.0090 (12)	0.0112 (13)
C2	0.0204 (13)	0.0237 (14)	0.0226 (13)	−0.0014 (10)	−0.0004 (10)	0.0048 (10)
C3	0.0238 (13)	0.0166 (13)	0.0239 (13)	0.0003 (10)	0.0028 (10)	−0.0028 (10)
C4	0.0368 (17)	0.0264 (16)	0.0526 (19)	−0.0028 (13)	0.0048 (14)	−0.0181 (14)
C5	0.0220 (13)	0.0188 (13)	0.0240 (13)	−0.0014 (10)	0.0032 (10)	−0.0006 (10)
C6	0.0268 (15)	0.0261 (15)	0.0279 (14)	−0.0022 (11)	−0.0013 (11)	−0.0015 (11)
C7	0.0353 (15)	0.0160 (13)	0.0245 (14)	−0.0001 (11)	0.0018 (11)	−0.0036 (10)
C8	0.0314 (16)	0.0176 (14)	0.0533 (19)	0.0005 (11)	0.0024 (14)	−0.0028 (13)
N1	0.0238 (11)	0.0189 (11)	0.0204 (11)	−0.0009 (9)	0.0024 (9)	−0.0018 (8)
N2	0.0233 (11)	0.0194 (11)	0.0265 (11)	−0.0018 (9)	0.0059 (9)	−0.0022 (9)
O2	0.0224 (10)	0.0265 (10)	0.0264 (10)	0.0019 (8)	0.0089 (8)	−0.0021 (8)
O3	0.0257 (10)	0.0273 (11)	0.0241 (9)	0.0011 (8)	−0.0006 (8)	−0.0131 (8)
O4	0.0299 (10)	0.0184 (9)	0.0216 (9)	0.0015 (8)	−0.0069 (8)	0.0001 (7)
O5	0.0279 (10)	0.0307 (11)	0.0182 (9)	−0.0086 (8)	−0.0007 (7)	0.0056 (7)
O6	0.0364 (11)	0.0165 (9)	0.0243 (9)	−0.0111 (8)	0.0055 (8)	−0.0005 (7)
O7	0.0240 (9)	0.0152 (9)	0.0320 (10)	0.0037 (7)	−0.0057 (8)	0.0033 (7)
O8	0.0270 (10)	0.0154 (9)	0.0292 (10)	−0.0076 (7)	0.0021 (8)	−0.0041 (7)
O9	0.0253 (10)	0.0245 (10)	0.0241 (9)	0.0102 (8)	0.0011 (7)	−0.0008 (7)
P1	0.0174 (3)	0.0172 (3)	0.0146 (3)	−0.0003 (2)	0.0020 (2)	−0.0026 (2)
P2	0.0178 (3)	0.0106 (3)	0.0220 (3)	0.0003 (2)	0.0008 (2)	0.0001 (2)

### Geometric parameters (Å, º)

**Table:**

Al1—O2	1.7215 (18)	C7—C8	1.505 (4)
Al1—O9	1.7251 (17)	C7—H7A	0.9700
Al1—O4^i^	1.7300 (17)	C7—H7B	0.9700
Al1—O6^ii^	1.7326 (18)	C8—H8B	0.9700
C1—C3	1.540 (3)	C8—H8A	0.9700
C1—H1D	0.9600	N1—H1A	0.8900
C1—H1E	0.9600	N1—H1B	0.8900
C1—H1F	0.9600	N1—H1C	0.8900
C2—N1	1.491 (3)	N2—H2A	0.8900
C2—C3	1.530 (3)	N2—H2B	0.8900
C2—C8	1.538 (4)	N2—H2C	0.8900
C2—H2	0.9800	O2—P1	1.5315 (17)
C3—C4	1.529 (3)	O3—P1	1.4908 (17)
C3—C5	1.557 (3)	O4—P1	1.5329 (16)
C4—H4A	0.9600	O4—Al1^i^	1.7300 (17)
C4—H4B	0.9600	O5—P1	1.5519 (18)
C4—H4C	0.9600	O5—H5B	0.9600
C5—N2	1.509 (3)	O6—P2	1.5458 (17)
C5—C6	1.529 (3)	O6—Al1^ii^	1.7326 (18)
C5—C7	1.539 (3)	O7—P2	1.5359 (16)
C6—H6A	0.9600	O8—P2	1.4986 (17)
C6—H6B	0.9600	O9—P2	1.5446 (17)
C6—H6C	0.9600		
			
O2—Al1—O9	108.34 (9)	C8—C7—C5	105.8 (2)
O2—Al1—O4^i^	110.23 (9)	C8—C7—H7A	110.6
O9—Al1—O4^i^	109.95 (9)	C5—C7—H7A	110.6
O2—Al1—O6^ii^	110.69 (9)	C8—C7—H7B	110.6
O9—Al1—O6^ii^	109.18 (9)	C5—C7—H7B	110.6
O4^i^—Al1—O6^ii^	108.44 (9)	H7A—C7—H7B	108.7
C3—C1—H1D	109.5	C7—C8—C2	105.8 (2)
C3—C1—H1E	109.5	C7—C8—H8B	110.6
H1D—C1—H1E	109.5	C2—C8—H8B	110.6
C3—C1—H1F	109.5	C7—C8—H8A	110.6
H1D—C1—H1F	109.5	C2—C8—H8A	110.6
H1E—C1—H1F	109.5	H8B—C8—H8A	108.7
N1—C2—C3	116.6 (2)	C2—N1—H1A	109.5
N1—C2—C8	110.1 (2)	C2—N1—H1B	109.5
C3—C2—C8	105.3 (2)	H1A—N1—H1B	109.5
N1—C2—H2	108.2	C2—N1—H1C	109.5
C3—C2—H2	108.2	H1A—N1—H1C	109.5
C8—C2—H2	108.2	H1B—N1—H1C	109.5
C4—C3—C2	112.2 (2)	C5—N2—H2A	109.5
C4—C3—C1	108.9 (2)	C5—N2—H2B	109.5
C2—C3—C1	110.7 (2)	H2A—N2—H2B	109.5
C4—C3—C5	114.2 (2)	C5—N2—H2C	109.5
C2—C3—C5	98.78 (19)	H2A—N2—H2C	109.5
C1—C3—C5	111.8 (2)	H2B—N2—H2C	109.5
C3—C4—H4A	109.5	P1—O2—Al1	152.61 (12)
C3—C4—H4B	109.5	P1—O4—Al1^i^	148.27 (12)
H4A—C4—H4B	109.5	P1—O5—H5B	109.2
C3—C4—H4C	109.5	P2—O6—Al1^ii^	140.61 (11)
H4A—C4—H4C	109.5	P2—O9—Al1	140.31 (11)
H4B—C4—H4C	109.5	O3—P1—O2	112.02 (10)
N2—C5—C6	106.61 (19)	O3—P1—O4	109.53 (10)
N2—C5—C7	107.00 (19)	O2—P1—O4	109.11 (10)
C6—C5—C7	112.0 (2)	O3—P1—O5	110.98 (10)
N2—C5—C3	113.7 (2)	O2—P1—O5	106.98 (10)
C6—C5—C3	112.7 (2)	O4—P1—O5	108.10 (9)
C7—C5—C3	104.73 (19)	O8—P2—O7	113.33 (10)
C5—C6—H6A	109.5	O8—P2—O9	111.06 (10)
C5—C6—H6B	109.5	O7—P2—O9	107.29 (9)
H6A—C6—H6B	109.5	O8—P2—O6	108.91 (10)
C5—C6—H6C	109.5	O7—P2—O6	108.08 (10)
H6A—C6—H6C	109.5	O9—P2—O6	108.00 (10)
H6B—C6—H6C	109.5		

Symmetry codes: (i) −*x*+1, −*y*+1, −*z*+2; (ii) −*x*, −*y*+1, −*z*+2.

### Hydrogen-bond geometry (Å, º)

**Table:**

*D*—H···*A*	*D*—H	H···*A*	*D*···*A*	*D*—H···*A*
N1—H1*A*···O3	0.89	1.87	2.751 (2)	168
N1—H1*B*···O5^iii^	0.89	2.06	2.910 (3)	158
N1—H1*C*···O8^iv^	0.89	1.85	2.709 (3)	162
N2—H2*A*···O3^v^	0.89	2.00	2.858 (2)	160
N2—H2*B*···O8^ii^	0.89	2.14	3.010 (2)	167
N2—H2*C*···O7^vi^	0.89	1.89	2.766 (3)	169
O5—H5*B*···O7^vii^	0.96	1.59	2.498 (2)	156

Symmetry codes: (ii) −*x*, −*y*+1, −*z*+2; (iii) −*x*+1, −*y*+1, −*z*+1; (iv) −*x*+1/2, *y*−1/2, −*z*+3/2; (v) *x*−1, *y*, *z*; (vi) −*x*−1/2, *y*−1/2, −*z*+3/2; (vii) *x*+1, *y*, *z*.
